# Harnessing precision nutrition to individualize weight restoration in anorexia nervosa

**DOI:** 10.1186/s40337-025-01209-x

**Published:** 2025-02-17

**Authors:** Isabel Rodriguez, Laura M. Huckins, Cynthia M. Bulik, Jiayi Xu, Daria Igudesman

**Affiliations:** 1 School of Medicine, University of North Carolina at Chapel Hill, Chapel Hill, NC USA; 2 Department of Psychiatry, Yale University School of Medicine, New Haven, CT USA; 3 Department of Nutrition, University of North Carolina at Chapel Hill, Chapel Hill, NC USA; 4 Department of Psychiatry, University of North Carolina at Chapel Hill, Chapel Hill, NC USA; 5 Department of Medical Epidemiology and Biostatistics, Karolinska Institutet, Stockholm, Sweden; 6 AdventHealth Translational Research Intsitute, 301 E Princeton St, Orlando, FL 32805 USA

**Keywords:** Anorexia nervosa, Precision nutrition, Weight restoration, Gut microbiota, Nutrigenetics, Nutrigenomics, Nutritional psychiatry, Energy balance

## Abstract

Anorexia nervosa (AN) is a severe psychiatric disorder for which effective treatment and sustained recovery are contingent upon successful weight restoration, yet the efficacy of existing treatments is suboptimal. This narrative review considers the potential of precision nutrition for tailoring dietary interventions to individual characteristics to enhance acute and longer-term weight outcomes in AN. We review key factors that drive variation in nutritional requirements, including energy expenditure, fecal energy loss, the gut microbiota, genetic factors, and psychiatric comorbidities. Although scientific evidence supporting precision nutrition in AN is limited, preliminary findings suggest that individualized nutrition therapies, particularly those considering duration of illness and the gut microbiota, may augment weight gain. Some patients may benefit from microbiota-directed dietary plans that focus on restoring microbial diversity, keystone taxa, or functions that promote energy absorption, which could enhance weight restoration—although stronger evidence is needed to support this approach. Furthermore, accounting for psychiatric comorbidities such as depression and anxiety as well as genetic factors influencing metabolism may help refine nutrition prescriptions improving upon existing energy estimation equations, which were not developed for patients with AN. Given the reliance on large sample sizes, costly data collection, and the need for computationally intensive artificial intelligence algorithms to assimilate deep phenotypes into personalized interventions, we highlight practical considerations related to the implementation of precision nutrition approaches in clinical practice. More research is needed to identify which factors, including metabolic profiles, genetic markers, demographics, and habitual lifestyle behaviors, are most critical to target for individualizing weight restoration, and whether personalized recommendations can be practicably applied to improve and sustain patient recovery from this debilitating disorder with high relapse and mortality rates.

## Abstract (plain language summary)

Anorexia nervosa (AN) is a serious mental health condition for which successful treatment and recovery depend on restoring weight to a healthy level. Current treatments often fall short of being effective. This review examines the potential of precision nutrition, which tailors dietary plans to individual characteristics, to improve weight outcomes and promote long-term recovery in AN while minimizing psychological and physiological discomfort during treatment. We explore various factors that influence nutritional needs in AN and may therefore be used to tailor dietary prescriptions, including loss of dietary energy in stool, gut microorganisms inhabiting the colon, genetic differences, and mental health conditions co-occurring with AN. Although there is limited evidence supporting the use of precision nutrition in AN, early research suggests that nutrition therapies personalized to AN duration may improve weight gain. More recent precision nutrition designs consider not just one or a few factors, but aggregate numerous patient factors including biology, personal preferences, and sociodemographics to find the optimal diet with the aid of artificial intelligence. Before precision nutrition therapies can be implemented in clinical practice, extensive research needs to be conducted in AN, and practical considerations related to the implementation of precision nutrition in clinical care must be addressed.

## Introduction

### Background

#### Overview of anorexia nervosa (AN)

Anorexia nervosa (AN) is a severe psychiatric disorder characterized by extreme low weight, intense fear of gaining weight, and an inability to recognize the severity of illness [1]. It often leads to severe malnutrition resulting in a host of medical complications that can be fatal if not properly treated [2]. AN is a challenging illness to treat [3, 4], with high relapse and mortality rates due to an interplay of biological, psychological, and social factors including genetic predisposition, limited access to evidence-based treatment, and the persistence of cognitive distortions related to weight, body image, and food after weight restoration [5–7]. 

#### Significance of weight restoration in AN

Weight restoration (also termed nutritional rehabilitation, clinical refeeding, or renourishment) is widely recognized as a crucial first step in treatment [8, 9]. This initial intervention is necessary for normalizing body functions and provides a foundation to address the psychological aspects of the disorder. Clinically, discharging patients closer to their target weight reduces the risk of relapse [10]. Despite the importance of weight restoration in AN treatment, consensus is lacking on optimal renourishment strategies to achieve weight gain while minimizing physiological and psychological sequelae such as gastrointestinal discomfort, refeeding syndrome, and psychiatric distress. Furthermore, treatments are sub-optimally effective as exemplified by relapse rates as high as 50% [10, 11] and may need to be tailored to the individual to maximize long-term efficacy [3]. 

#### Time for a precision approach in the treatment of AN?

AN etiopathology is complicated and heterogeneous, encompassing environmental and societal risk factors alongside metabo-psychiatric risk factors including genetic variation and gut microbial taxa [12]. The relative roles of these disparate risk factors likely change between patients and may interact to increase risk. Precision approaches may allow tailoring of treatment decisions to individual risk factors and disease characteristics. For example, researchers investigating other psychiatric conditions [13] and chronic diseases such as diabetes (type 1, 2, and monogenic forms) [14] and obesity [15] have begun to explore whether aggregating indicators of biological variability can help to explain interindividual responses to the same treatment (i.e., precision medicine [16] and relevant to the present review, precision nutrition).

Precision nutrition refers to the stratification of dietary recommendations based on individual factors from data sources such as genetics, microbiota profiles, environmental factors, and phenotypic data to optimize health outcomes [17]. Early indications suggest an enhanced efficacy of artificial intelligence (AI)-assisted precision nutrition approaches relative to standard-of-care for minimizing postprandial glucose excursions [18]. In contrast, no studies focused on patients with AN have used AI to optimize nutrition therapies for weight restoration based on deep phenotyping, or the comprehensive collection of personal biological data that facilitates tailoring nutrition recommendations based on genomics and other data. This gap in scientific knowledge may stem from AN research being underfunded and concerns that algorithmically derived diet prescriptions may reinforce cognitive rigidity around eating. We caution that the use of AI may raise ethical concerns including data privacy and algorithmic bias. For example, given the historic focus on White, female patients with AN [19], it is possible that derived algorithms will prioritize factors relevant to these groups, rather than creating broadly applicable algorithms, ultimately leading to outputs that systematically disadvantage underserved groups [20]. These biases may, for example, result in algorithms that fail to account for differences in dietary preferences or the utility of BMI across races, or that do not account for differences in body composition across sexes.

Accordingly, the objective of this narrative review is to evaluate the scientific evidence investigating factors that can be used to personalize dietary interventions for optimizing weight restoration—an established benchmark of AN recovery that predicts future relapse [10]. We note that we use the term “personalized” interchangeably with “precision” in the context of this review, as previously suggested [17], given that "personalized" recommendations will never be so granular as to apply to a single individual but rather reflect AI-informed subgroup stratification. We first describe existing weight restoration approaches and their limitations, followed by a review of how energy expenditure, fecal energy loss, the gut microbiota, genetic factors, and psychiatric comorbidities could be used to personalize and improve the long-term efficacy of treatments targeting weight restoration. We additionally highlight gaps in scientific evidence that may be useful to address for advancing clinical practice. We acknowledge that standardized and comprehensive definitions of recovery are lacking [21] and emphasize the need for future studies to address components of recovery other than weight restoration.

## Current approaches to weight restoration in AN

### Traditional nutrition interventions

#### Current nutritional related best practices in inpatient and residential AN treatment

Traditional nutrition interventions to restore weight in AN typically followed a “start low and go slow” approach, beginning with meal plans under ~ 1,200 calories and gradually increasing intake to mitigate the risk of refeeding syndrome [22–24]. Refeeding syndrome is a potentially fatal medical complication that encompasses severe electrolyte and fluid shifts due to rapid spikes in endogenous insulin when calories and nutrients are reintroduced to malnourished patients too rapidly [25]. Recent research shows that a conservative low-calorie method is associated with decreased weight gain and prolonged hospital stays in patients with AN [22]. Emerging evidence supports higher initial caloric prescriptions combined with close medical monitoring and electrolyte replacement to prevent refeeding syndrome [26]. 

Although there is no consensus on the ideal initial calorie prescription, with recommendations ranging from 1,000 to 2,400 kcal per day, contemporary guidelines lean towards a more aggressive nutritional approach [8, 9, 27–30]. Once the target calorie level is reached, adjustments are made based on the rate of weight gain to promote weight increases of 2 to 4 lbs (i.e., 0.9 to 1.8 kg) per week [8]. Notably, until recently few randomized controlled trials were available on refeeding strategies, so recommendations were largely based on clinical experience [31]. 

Inpatient targets for weight restoration center on discharge criteria, which typically require patients to achieve a weight threshold that ensures medical stability and supports continued recovery. The target weight is often set at ~ 85% of individually determined ideal body weight, although there is variability in how this target is calculated [8, 29]. For adults, a body mass index (BMI) of 20 kg/m^2^ is sometimes used as an initial guide, considering other factors such as premorbid weight, normalization of eating patterns, and medical stability [8]. More research is needed to determine if certain patient characteristics warrant a more or less aggressive refeeding approach (i.e., a precision approach). Most studies have focused on predominantly non-Hispanic White adolescents and young adults [22, 24, 32–35], limiting generalizability to more diverse populations—a point that is imperative to address when developing precision nutrition therapies.

#### Macronutrient and micronutrient targets

Although the primary focus of this review is on weight restoration through appropriately addressing calorie needs, we recognize that other dietary factors have the potential to impact long-term weight restoration both through indirect (i.e., personal preference and adherence, and gastrointestinal side effects) and direct mechanisms (i.e., influence on fat oxidation and thus body weight). Thus, we briefly review current guidelines for macronutrient distribution and micronutrient targets in AN treatment. Overall, guidance for macronutrient composition aligns with those recommended for adults without an eating disorder (ED). The recommended composition includes 50–55% of total energy intake from carbohydrates, 25–30% from fats, and 15–20% from proteins [9]. Some studies have indicated that macronutrient distribution, rather than calories alone, can influence the risk of refeeding syndrome. Specifically, a higher carbohydrate intake has been linked to an increased risk of post-prandial hypoglycemia as well as an increased risk of refeeding hypophosphatemia [31, 36]. Prescriptions for both dietary fat and fiber are routinely individualized throughout treatment [9] to address psychological challenges including dietary fat aversion and physiological barriers including constipation. In some cases, as patients progress through treatment and their caloric prescription increases, they can benefit from having calorically dense dietary fat incorporated into their meals to avoid significant increases in food volume.

Micronutrient management during nutrition rehabilitation targets the prevention of refeeding syndrome, correcting deficiencies resulting from malnutrition, and supporting metabolic processes including energy metabolism. However, guidelines do not provide recommendations for micronutrient supplementation or are inconsistent regarding the use of nutrients such as thiamine, vitamin D, and calcium, among others [9, 27, 29, 30]. Whether micronutrient supplementation can augment weight restoration is an open question. A small study enrolling 35 female patients with AN suggests that zinc supplementation may enhance the rate of BMI increase compared to placebo; [37] however, current evidence is too limited to make firm conclusions. Micronutrient supplementation should always be individualized and could be considered in AI-assisted precision nutrition algorithms for weight restoration.

### Limitations of these approaches and related challenges

#### Appropriateness of equations for estimating energy requirements during weight restoration

Target calorie levels are determined using estimated resting metabolic rate (RMR). Defined as energy expenditure at complete rest, RMR represents energy requirements for maintaining vital bodily functions and comprises 60-75% of 24-hour energy expenditure [38]. Most equations use sex, weight, height, and age to approximate RMR, which is multiplied by an activity factor to estimate total daily energy requirements. Although the Nutrition Care Manual recommends estimating RMR for patients with AN using the Mifflin St. Jeor equation [9], in practice and in research a variety of equations including Harris Benedict are used [39]. With the exception of the Schebendach correction, equations used in research and clinical practice were developed in generally healthy populations [39] and have not been adapted for use in patients with underweight such as those with AN, which is a major limitation. Numerous hormonal and parasympathetic mechanisms governing energy metabolism are dampened in AN to lower metabolic rate and conserve vital energetic processes [40], and virtually all equations [41] overestimate RMR in patients with AN [39]. Energy needs were overestimated by an average of ~ 375 kilocalories/day (i.e., ~ 45%) in 43 adolescent girls with AN and by ~ 200 kilocalories/day in 194 adult women with AN aged 18–40 when comparing the Harris Benedict and World Health Organization/ Food and Agriculture Organization / United Nations University equations against indirect calorimetry [39]. Correcting the Harris-Benedict equation with the Schebendach method for patients with AN improved accuracy in adolescents, but not adults, with the amount of bias dependent on individual characteristics such as age and BMI [39]. Although it would seem that overestimating energy requirements could be advantageous in AN treatment outside of the acute risk of refeeding syndrome, greater precision is required to set accurate treatment targets, enable more facile medical management, and reduce psychological and physiological distress associated with rapid weight gain and bloating.

#### Low long-term efficacy

Although achieving weight restoration during treatment is a critical component of recovery, weight maintenance, or maintaining a healthy weight over time, is an essential component of sustained recovery [4]. By this definition, the long-term efficacy of inpatient treatment is low particularly within the first year post-discharge [42] when relapse rates are as high as 50% [10, 11]. Although there is no widely accepted definition for relapse, it is often defined as a BMI <18.5 kg/m^2^ or a body weight loss ≥ 15% [3]. The low efficacy of AN treatment can lead to a cycle in which patients temporarily regain weight during treatment only to lose it shortly after discharge, leading to a “revolving door” [11]. This strongly points to the need for novel strategies addressing both physiological and psychological barriers to maintenance of restored weight. Targeting these efforts towards subsets of patients most likely to relapse following inpatient care could be economically and clinically efficient. For example, a meta-analysis of 28 studies found that over 31 months of follow-up, of the 37% of patients with AN who relapsed, those with higher pre-treatment depression and lower post-treatment BMI were more likely to relapse [43]. The study authors concede that larger sample sizes are needed to reach consensus about other predictors of relapse including AN duration, age, sex, comorbidities, and personality [43]—all of which could be tested in precision nutrition approaches.

#### Physiological and psychological distress

For weight restoration protocols to be maximally effective in the long-term, treatments must consider both the psychological and physiological sequelae of refeeding, which are distressing for patients and unique to each individual. In qualitative studies, participants consistently reported feeling that the psychological aspect of treatment was underemphasized in the inpatient setting [44]. For example, patients experience heightened anxiety, negative emotions, and increased ED cognitions during meals [45]. Additionally, individuals with AN often report postprandial gastrointestinal discomfort, including abdominal pain, bloating, and nausea. Impaired gut motility has been identified as a complication of AN which likely contributes to these symptoms [46]. It has also been hypothesized that prolonged starvation leads to gut dysfunction, further contributing to gastrointestinal symptoms. Although medical nutrition therapy is personalized to some degree, this is done based on a limited number of factors (e.g., sex, weight, height, and age) to predict energy requirements [9] and in a trial-and-error fashion to adjust calories when treatment response proves to be suboptimal. Thus, an opportunity exists to identify additional predictors that can be used to tailor nutrition therapies *before* treatment commences, accelerating weight restoration while minimizing distress and reducing the likelihood of relapse.

### Potential predictors of weight restoration in the inpatient treatment of AN: overview

A fundamental gap in AN treatment that precision nutrition paradigms could address is whether it is possible to accelerate weight restoration within higher levels of care while minimizing discomfort and promoting long-term recovery by tailoring various aspects of dietary intake to deep phenotypic traits. Proposed traits include various sources of -omics data, discussed in greater detail in the sections that follow. We highlight traits that may have high importance for weight restoration and longer-term recovery, including energy expenditure, fecal energy loss, the gut microbiota, genetics, and psychiatric comorbidities. While intentional food restriction is often assumed to underlie post-treatment weight loss and relapse, it is important to consider that physiological factors such as a perturbed gut microbiota are also hypothesized to reinforce negative energy balance in AN in a non-volitional manner [47]. We recognize that numerous other sociodemographic and clinical characteristics are potentially important to consider when testing precision nutrition protocols against one-size-fits-all approaches and allude to these factors in the below section titled ”Practical considerations for implementation”. Diet-related variables on which to tailor treatments could include initial caloric prescriptions, rates of caloric increase, macronutrient distributions, the timing or frequency of meals, and micronutrient supplementation. Though we focus our attention on calorie prescriptions given our emphasis on weight restoration, other dietary components are explored as they may further enhance treatment outcomes.

### Variation in energy expenditure across patients and throughout recovery

Although traditional equations overestimate energy requirements in patients with AN at the beginning of treatment due to adaptive mechanisms of energy conservation, many patients are resistant to weight gain once refeeding is initiated [41]. This so-called “rebound hypermetabolism” is the proposed phenomenon of increased energy expenditure during refeeding in AN beyond that predicted by gains in fat-free mass alone. A recent scoping review of 36 studies published between 1984 and 2020 did not find strong evidence to support this phenomenon, suggesting alternate explanations could be at play [41]. Alternatively, it is possible that group-level averages obscure rebound hypermetabolism in select subgroups, although evidence to support this notion is lacking.

Irrespective of whether hypermetabolism occurs, it is evident that existing equations developed for use in generally healthy populations do not perform well in patients with AN and should be adapted to disease-related characteristics. One study consisting of 138 women in outpatient treatment for AN found improved performance of the Schebendach equation when accounting for AN duration (< 3 years or ≥ 3 years) and subtype (restrictive AN or binge-purge AN) [48]. The development of such equations represents a potential application for precision nutrition, utilizing data from indirect calorimetry to generate equations that more reliably estimate energy requirements in patients with AN.

Additionally, caloric intake must be further calibrated to match the increased energy expenditure that typically occurs in the transition from inpatient care to a free-living environment. Evidence for tailoring calorie and nutrient targets post-discharge according to individual characteristics is lacking, although personalized strategies are generally recommended [9]. It is possible that wearable devices such as accelerometers that are often factored into precision nutrition paradigms could be useful for periodic tracking of energy expenditure during outpatient recovery from AN. Such tools could support relapse prevention by allowing providers to iteratively personalize meal plans when detecting compulsive exercise or other movement patterns that increase energy requirements. This strategy could be particularly useful for athletes in recovery and falls within the bounds of current guidelines for outpatient nutrition monitoring, which should be done weekly or biweekly for up to a year or longer to prevent relapse [9]. Importantly, the focus of these approaches should remain on equipping providers with practical tools and reliable data to inform nutritional interventions rather than promoting calorie tracking by patients.

### Fecal energy loss

Another potential source of inaccuracy in estimated energy requirements is fecal energy loss. It is hypothesized that incomplete energy absorption results from an impaired capacity of the gut to absorb nutrients in AN, which is ostensibly restored following treatment, although studies to support this theory in humans are scarce. In 12 participants *without* an ED, underfeeding relative to energy requirements increased relative fecal energy loss (i.e., per gram of stool) compared to an overfeeding condition [49]. Similarly, a recent report found that patients with AN excreted more fecal energy pre-treatment (3.75 ± 0.73 kcal per gram of stool) compared to post-treatment (1.71 ± 0.26 kcal per gram of stool) and compared to controls without AN (2.51 ± 0.33 kcal per gram of stool) [50]. Of note, pre-treatment standard deviation in stool energy was more than twice that of post-treatment or control values [50]. This suggests there may be subgroups of individuals for whom fecal energy loss is more pronounced during acute AN. These individuals may require higher calorie requirements upon admission, along with strategies to restore gastrointestinal health, and/or pre- and probiotic supplements to augment dietary energy absorption. Future studies should measure *absolute* fecal energy loss (i.e., kilocalories/24 h) while accounting for precise estimates of energy intake and energy expenditure. This will elucidate whether the impact of fecal energy loss on weight restoration is likely to be clinically meaningful, and in which types of patients.

### Gut microbiome

Prevailing hypotheses posit that fecal energy loss is a primary mechanism by which the gut microbiota—the vast ecosystem of microbes residing in the intestinal tract—modulates weight in AN. In theory, a nutrient-poor environment disrupts microbial community structure and damages intestinal epithelial integrity during prolonged starvation [51]. These alterations could hinder nutrient absorption in the small intestine and reduce colonic microbial energy extraction from undigested food components in the colon [51], exacerbating weight gain resistance in AN. Another potential role for precision nutrition could therefore center on restoring gut functionality through individualized diet interventions that augment key microbial activities. For example, fermentable dietary fibers increase microbial production of short-chain fatty acids, which promote gut barrier integrity and increase overall energy availability to the human host [52]. Short-chain fatty acids are deficient in patients with AN, and may therefore serve as a microbiota-focused target for weight restoration in some patients [53]. If not normalized during treatment, it is posited that a gut microbiota adapted to nutrient insufficiency could perpetuate negative energy balance and precipitate relapse, which is notably independent of willful energy restriction by the patient [47]. This hypothesis requires confirmation in experimental studies.

The majority of microbiome-focused studies in AN have been observational and were essential for generating hypotheses about mechanisms of AN pathology. These initial observations describe alterations in the diversity, composition, and function of the gut microbiota in patients with AN compared to controls, or pre- and post-treatment of AN, and have been reviewed in detail [53–55]. Far fewer studies have directly tested the contribution of the gut microbiota to weight restoration. A recent study combining epidemiological modeling of human data and experimental validation in a germ-free murine model revealed that serum metabolites mediate associations between the gut microbiota and ED-related psychiatric traits [55]. Fecal microbiota transplantation (FMT), which involves transferring fecal microbial communities from one organism to another, showed that small cohorts (*n* = 6 to 8) of calorically restricted germ-free mice inoculated with the stool of three patients with AN gained weight more slowly over three weeks relative to mice that received stool from control donors without AN [55]. This coincided with group differences in appetite-related genes in the hypothalamus. An earlier study utilizing FMT from four AN donors pre- and post-treatment to much larger groups of mice (*n* = 50 to 53) [56] suggested no differences in weight gain four weeks after colonization among mice receiving FMT from AN donors pre- or post-treatment or four control donors without AN, but these mice were not fed a calorie-restricted diet. FMT has been tested in only two published case reports in patients with AN [57, 58], where weight restoration was improved in one case but not the other, which limits making conclusions. Outside of FMT, direct modulation of microbial communities and their ability to shunt energy from nondigestible foodstuffs to the human host can be achieved through targeted diet interventions [49] and may therefore represent a safer route for investigating personalized microbiota-focused therapies for weight restoration, particularly among patients who are medically compromised.

Indeed, emerging scientific evidence from rigorously designed clinical trials indicates that microbes play a clinically meaningful role in human energy balance and that this host-microbe interaction can be modulated through diet [49, 59]. Despite its lower caloric density, a microbiota-directed complementary food prototype containing chickpea flour, soy flour, peanut flour, and banana accelerated the rate of increase in weight-for-age and weight-for-length z-scores relative to a standard ready to eat complementary food in 123 Bangladeshi children with moderate acute malnutrition [59]. This underscores the need to look beyond calories accessible by human small intestinal enzymes and towards nondigestible carbohydrates and amino acids that may be transformed by colonic microbes into viable energy sources for the human host. In this same study, changes in 21 bacterial taxa were positively correlated with the weight-for-age z-score [59]. The authors recently characterized potential mechanisms in these same stool samples and found that genomic potential of the bacterium *Prevotella copri* to degrade glycans, a carbohydrate constituent of the microbiota-directed complementary food, may be responsible for the effects [60]. However, the prevalence of this microbe is dwindling in Western populations, suggesting that microbiota-focused therapies should be matched to an individual’s geographic milieu and baseline microbiome composition to support engraftment of target microbes [61]. It is likely that the effects of diet-microbiome interactions on weight and gut-related factors such as bloating vary across individuals and interact with other host characteristics, calling for a precision nutrition approach [62]. Although we focus on whole diet-microbiome interactions in this review, we acknowledge that precision probiotics, or tailoring probiotics to an individual’s unique microbial ecosystem, may be a future avenue to improve weight restoration in combination with whole food approaches [63]. 

### Psychiatric comorbidities and nutritional psychiatry

Beyond the period of acute inpatient weight restoration, tailoring ongoing nutritional rehabilitation treatments to the psychiatric comorbidities of AN, including depression, anxiety disorders, obsessive compulsive disorder, and substance use disorders [64] could help to maintain restored weight and prevent relapse [65]. This premise is rooted in the concept of nutritional psychiatry, which highlights the importance of macronutrients and micronutrients in the synthesis and metabolism of hormones and neurotransmitters governing eating behavior, emotional regulation, and cognition [66]. The evidence for macronutrient modulation has been focused on low carbohydrate and ketogenic diets to treat mood and anxiety disorders due to their anxiolytic effects in rodent models [67]. A systematic review of 12 human interventions to treat mood and anxiety disorders found that there was insufficient high-grade evidence to support the use of these diets despite an initial signal of effectiveness for those with bipolar disorder, schizoaffective disorder, and depression/anxiety [68]. In AN patients with comorbid depression and anxiety, the risk of promoting restrictive eating behaviors may, however, outweigh the potential benefit of carbohydrate-restricted diets.

The treatment of major depressive disorder with nutritional supplements including omega-3 fatty acids, vitamin D, probiotic strains, and zinc is now supported by the World Federation of Societies of Biological Psychiatry [69] with the use of omega-3 fatty acids additionally recommended by the International Society for Nutritional Psychiatry Research [70]. The evidence on omega-3 fatty acids is particularly strong for those with dietary insufficiency, which could result from the characteristic dietary fat restriction seen in AN [71]. Proposed mechanisms conferring the benefits of omega-3s include dampening of chronic low-grade inflammation, resolution of oxidative stress, and enhanced neuroplasticity [70]. These hypotheses must be carefully tested as pharmacotherapies that treat depression are generally ineffective for resolving symptoms of AN [72]. It is unclear whether treating depressive symptoms in AN patients through nutritional means would target different pathways and be more effective [66]. Both antipsychotics and antidepressants can decelerate gut transit time [73] and may therefore have unique effects on gastrointestinal motility and gut microbiota composition relative to food-based therapies [74]. Indeed, microbial diversity is higher in patients who experience constipation with antipsychotics, which could reflect increased microbial fermentation that may be detrimental (i.e., if putrefactive fermentation of amino acids increases) [75]. Thus, nutrition therapies may offset some of the negative gut-related side-effects of these medications, minimizing discomfort during recovery.

The literature examining nutrient supplementation for the treatment of anxiety is largely focused on vitamin D, given its role in the regulation of serotonin synthesis and release [76]. A randomized controlled trial with 30 participants receiving standard of care treatment alone or standard of care plus weekly 50,000 IU of vitamin D supplementation showed significant improvements in anxiety symptoms and increased serotonin levels in the group receiving supplementation [77]. A meta-analysis including 15 observational studies of 927 participants found that 408 patients with AN had significantly lower serum levels of vitamin D before supplementation compared to 519 controls despite similar self-reported dietary intake levels of vitamin D between groups [78]. After vitamin D_3_ supplementation, serum vitamin D levels were normalized in patients with AN. Although it is unclear what proportion of patients had a frank deficiency at baseline and patients with AN tend to *over*report dietary intake relative to controls [79], this may suggest that consumption of vitamin D through food sources alone may not be sufficient to achieve normative serum levels in some patients with AN. Although not addressed in this paper, one explanation is that dietary fat restriction in AN reduces absorption of fat-soluble nutrients, which could increase vitamin D requirements. Given the high prevalence of vitamin D deficiency in the population of patients with AN [80] and its proposed role in mood regulation, supplementation could serve as a valuable adjunctive treatment for patients who present with low vitamin D levels. Further research is needed to determine the optimal dosage and duration of supplementation for improving both nutritional and psychological outcomes in patients with AN. High-quality trials are needed to determine whether tailoring whole diet approaches or nutritional supplements to the psychiatric comorbidities of AN can improve long-term outcomes by reducing risk factors for relapse including weight loss and comorbid symptoms.

### Genetic factors

Individual genetic differences could also contribute to the heterogeneity in weight restoration during treatment and subsequent risk of relapse. Human genome-wide association studies (GWAS) have identified genetic variants associated with AN [7]. Intriguingly, the same genetic variants are also inversely associated with cardiometabolic risk factors such as BMI [7]. However, no GWAS has yet investigated which genetic factors are linked to response to weight restoration treatment. Since nutritional rehabilitation is key for successful weight restoration for AN patients, it is possible that genetic factors associated with nutrient digestion, absorption, and metabolism [81–83] may modify treatment response.

Nutrigenetics and nutrigenomics are two sources of biological variability that could be integrated into the study of personalized nutrition for weight restoration. Whereas nutrigenetics is concerned with the effect of genetic factors on individual nutritional status, nutrigenomics examines the impact of nutrients on gene expression and downstream phenotypes [84]. A seminal nutrigenetic example of a genotype x diet interaction is the effect of *MTHFR* polymorphisms on blood folate concentration. Individuals with a TT genotype at the *MTHFR* 677 position had lower blood folate concentrations compared to those with a CC or CT genotype on a population level [85], suggesting that those with a TT genotype may require more dietary folate to maintain homeostatic levels of the nutrient.

Despite the critical role of diet in treating AN, few studies have investigated nutrigenetics in relation to AN. One potential area of interest is whether genetic variation in polyunsaturated fatty acid (PUFA) metabolism could be associated with aversion to high-fat food, a risk factor for AN relapse [86]. One gene candidate associated with PUFA metabolism is *EPHX2*, which encodes soluble epoxide hydrolase, a protein with catalytic functions on both n-3 and n-6 PUFAs [86, 87]. Given that variants in *EPHX2* have been implicated in AN risk [87], future research is needed to investigate the role of PUFAs in moderating *EPHX2* variant effects on dietary fat aversion and treatment response to weight restoration.

Nutrigenomic mechanisms are those by which dietary nutrients modify gene expression patterns in a tissue-specific manner through the addition or cleavage of methyl, acetyl, and other chemical groups to DNA or histones [88]. The effects of epigenetics can occur during “dietary transitions” (i.e., changes in dietary intake, such as during the development and recovery from AN) that may persist over years and induce shifts in gene expression [89]. Recent epigenome-wide association studies (EWAS) suggest this notion may extend to AN, finding that AN-induced DNA methylations in patients’ whole blood were reversible after treatment [90]. Further, the largest EWAS to date in 145 women with active AN found changes in DNA methylation on genes related to nutritional metabolism (e.g., lipids) compared to 49 women with one-year remission from AN and 64 controls with no ED [91]. However, it remains unknown whether these epigenetic changes were driven by specific changes in diet composition, weight gain itself, or other metabolic factors related to recovery. Additional questions that should be addressed include how these epigenetic alterations functionally affect gene expression and downstream phenotypes, and whether epigenetic markers are reversible in other tissues relevant to weight restoration such as the brain and the gut. Nonetheless, the nascent literature hints at the potential for epigenetic markers to serve as predictive biomarkers for AN recovery that may be useful to include in precision nutrition algorithms. Further study is required to elucidate whether dietary interventions can be harnessed to shift these epigenetic alterations towards a state that promotes weight restoration during recovery and prevents relapse long-term.

Another target for future study is the genetics of appetite and how it affects food intake to influence AN risk and relapse [92]. Food avoidant traits, such as satiety responsiveness, have been associated with slower weight gain throughout infancy and childhood [92]. The expression of circulating *POMC*, a gene involved in appetite suppression, was higher in patients with acute AN compared to AN patients who were weight restored [93]. One possibility is that the interaction of genetic factors with food intake affects appetite, which could modify susceptibility to relapse [7]. Since AN patients may have difficulties tuning into hunger and satiety cues, future studies on how personalized nutritional interventions can normalize interoceptive awareness of hunger and fullness may have important clinical value for patient prognosis and relapse prevention.

### Using artificial intelligence and deep phenotyping to optimize nutrition therapies

**Fig. 1:**
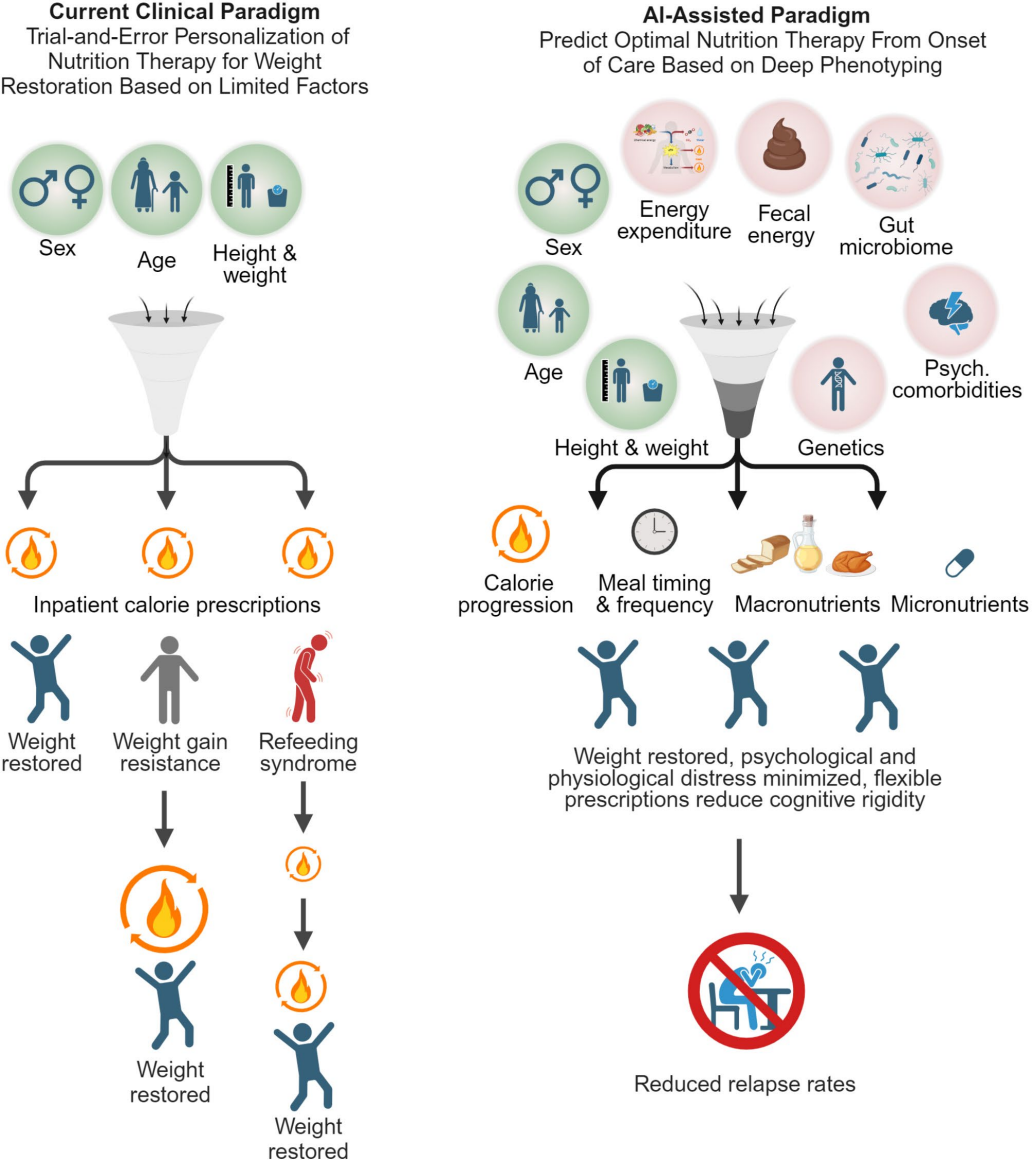
Proposed benefits of AI-assisted precision nutrition paradigms over standard-of-care nutrition therapies for weight restoration in AN

Current clinical paradigms determine BMI targets for treatment discharge and caloric targets for weight restoration based on limited factors including sex, age, height, and weight [9]. These crude predictions of BMI and caloric targets do not account for other interindividual differences such as genetics, gut microbiota composition, psychiatric and physiological comorbidities, and lifestyle behaviors such as habitual food intake and physical activity (Fig. 1). The integration of these factors using AI and machine learning (ML) approaches that learn from input data to make treatment predictions is one promising strategy to address treatment resistance and high relapse rates.

A comparison of current clinical practices and AI-assisted personalization for weight restoration highlights the evolution from trial-and-error approaches to more precise, data-informed practices. Factors currently considered are shown in green circles while those not currently considered are shown in red circles.

To our knowledge, only one study has applied ML to predict weight restoration in AN patients [94]. However, the authors only examined basic demographic, anthropometric, and psychological factors as input in their ML models and did not include any -omics data or other deep phenotypes. It is therefore unsurprising that the change in BMI from admission to discharge was the most important predictor of post-treatment BMI at 6 months. Although an important clinical implication is that it is critical for patients to restore sufficient weight during treatment before being discharged, this study did not address how to optimize inpatient weight restoration through diet using a comprehensive set of predictors [95]. 

### Practical considerations for implementation

#### Economics and cost effectiveness

**Fig. 2:**
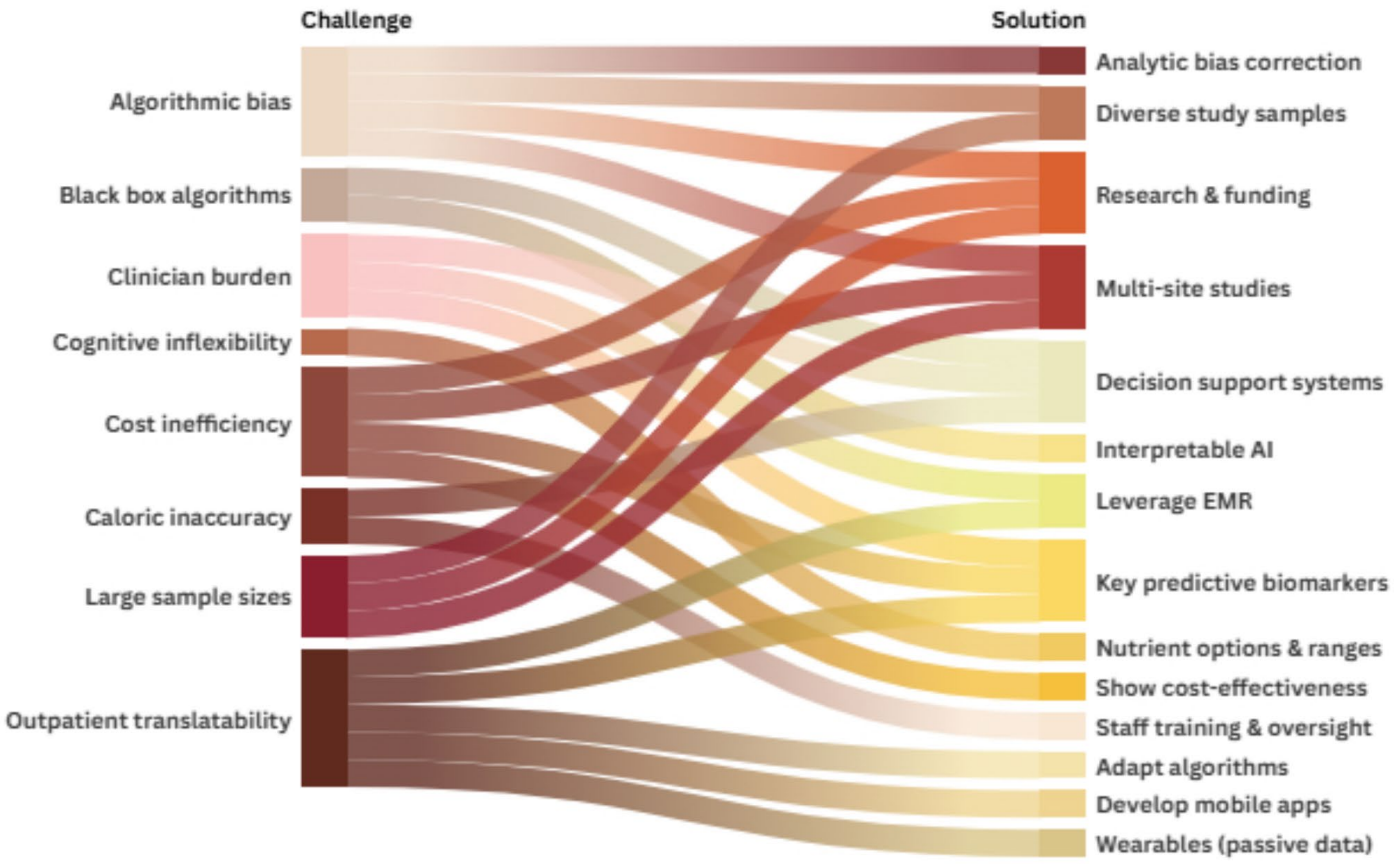
Challenges and proposed solutions for implementing precision nutrition in AN treatment

Formative research studies aiming to develop precision nutrition interventions collect large amounts of data to feed into AI algorithms. In this context, the utility of each biomarker must be considered. Although capturing an array of demographic, lifestyle, and multi-omic measurements may increase the predictive power of AI algorithms, this could increase costs and patient burden in the case of more invasive sampling (e.g., feces), particularly if repeated sampling is required to track biomarkers over the course of treatment. Thus, a key pragmatic question is whether a few target biomarkers of successful weight restoration can be identified to lower costs in a clinical setting (Fig. 2). Example biomarkers may include indicators of fecal energy loss, quantitative measurements of keystone microbial taxa associated with weight restoration [96], or pathway-specific genetic risk. Collection of blood and urine samples is less burdensome than fecal collection, so circulating and urinary biomarkers may need to be prioritized. Selection of biomarkers may also need to be tailored to individual characteristics, although research is needed to support this approach. Once the physiological basis for precision nutrition in AN is established, systematic cost effectiveness studies must be conducted to evaluate whether the benefits of personalized interventions justify the additional cost relative to standard-of-care.

Although data to support precision nutrition approaches for weight restoration in AN are limited, identifying solutions to implementation-related challenges and using them to inform future study designs will be key. Algorithmic bias—potential for biased predictions based on imbalanced cohorts used for training data (i.e., historic biases towards studies that primarily or uniquely include white, female AN patients); cognitive inflexibility—the risk of reinforcing cognitive rigidity through precise nutrition prescriptions in AN; analytic bias correction—mitigating the development of biased algorithms via rigorous algorithm design; and key biomarkers—identifying salient biomarkers of treatment response from the battery of phenotypes measured to reduce cost.

#### Bias and data limitations in AI algorithms

The use of AI algorithms in precision nutrition faces challenges related to bias and insufficient data, particularly in the context of EDs. Given the historical focus of ED research on white women, there is the risk of developing algorithms based on data that do not reflect the diverse presentation of EDs across various racial, ethnic, and gender groups. To improve the representation of diverse populations in ED studies, the ongoing Eating Disorders Genetics Initiative 2 aims to recruit 30% of ED cases from non-European populations by expanding its engagement with underrepresented communities [97]. In addition to recruiting more representative samples into AN studies, bias correction methods should be implemented. The concept of bias correction involves harnessing a range of analytic techniques to promote equity throughout the algorithm development process [98]. For example, reweighting training data by adjusting the importance of certain data points can help to ensure that historically underserved groups are adequately represented in the algorithm’s learning process.

### Patient acceptability

A proposed benefit of personalized nutrition interventions is their potential to enhance motivation for adhering to nutrition recommendations [99]. Whether this hypothesis has merit remains to be determined. A recent randomized trial testing a personalized omics-based Mediterranean diet against a standardized Mediterranean diet in 193 volunteers showed no differences in dietary adherence or health parameters over 21 weeks [100]. Another randomized study enrolling 347 participants representative of the US population showed improved subjective adherence to personalized compared to standardized dietary guidance for cardiometabolic health alongside lowered triglyceride levels, although wide variability in adherence was observed even in the personalized arm [101]. Although no such studies have been conducted in patients with AN, a foreseeable limitation is the risk of reinforcing cognitive rigidity in AN [102] through “precise” dietary recommendations. A potential remedy that is in line with current clinical practice is to provide patients with a variety of food options within the bounds of algorithmic suggestions. Importantly, patients should not be counseled to follow specific calorie or nutrient prescriptions, but instead receive guidance centered on flexible meal plans that are agnostic to calories. This could be achieved by personalizing existing frameworks such as the exchange system currently used in practice. It remains unclear if precision nutrition may compete with the development of behavioral flexibility, which should be investigated systematically.

#### Feasibility within existing systems of clinical care

Integrating precision nutrition into existing clinical care systems requires streamlined protocols, leveraging technology such as electronic medical record alerts to order tests and automate interpretation of results to facilitate implementation without overburdening clinicians. Interpretable ML algorithms with clear predictors of treatment response may be preferred to “black box” ML algorithms [103], which may undermine clinicians’ trust and are more complicated to debug during development [103, 104]. Once tailored recommendations are established, systems for improving the accuracy of calorie and nutrient provision and monitoring of intake in ED treatment centers must be instituted. Enhanced training of staff to monitor meal selection and completion is especially important when patients begin to self-portion their meals after reaching certain treatment milestones. Factoring in the goal of precision nutrition to prescribe the right diet to the right person *at the right time*, clinicians must also be cognizant that calorie and nutrient requirements may need to be recalculated over the course of treatment and updated prescriptions implemented with high fidelity.

### Implementing precision nutrition therapies in lower levels of care

Although most weight gain in AN treatment occurs in the inpatient setting, inpatient weight gain predicts sustained recovery over time [94]. Thus, by enhancing weight gain during inpatient care, precision nutrition has the potential to impact not only initial weight trajectories but also success in outpatient settings. Furthermore, personalized therapies may foster greater engagement with treatment [105, 106] and better prepare patients for the transition to outpatient settings. This hypothesis requires testing and translation within outpatient settings where the focus would shift from weight restoration to weight maintenance, which is a critical component of sustained recovery.

Previous randomized controlled trials suggest that personalized nutrition recommendations improve participant adherence relative to one-size-fits-all approaches [105–107]. While promising, whether this translates to ongoing outpatient treatments for AN warrants further investigation, particularly as the intensity of care and clinician support decrease. Algorithms will likely need to be trained on increased energy requirements and other factors unique to the outpatient setting, with even greater flexibility around food choice. Modern technologies such as mobile applications [108] may help to address primary barriers such as adherence to personalized recommendations by connecting patients with Registered Dietitians during all stages of the recovery process. Critically, clinicians should avoid providing quantitative dietary prescriptions to patients and instead translate recommendations into qualitative guideposts that patients can learn to implement independently.

### Future directions

The scientific evidence supporting precision nutrition interventions for weight restoration in AN is extremely limited. To our knowledge, there is one ongoing trial aimed at characterizing the feasibility, acceptability, and initial clinical efficacy of precision psychiatry treatment for ED symptoms compared to standard treatment, albeit neither nutrition nor weight restoration are addressed (NCT06208605). The study plans to enroll 320 adults aged 18–65 with any ED diagnosis except avoidant/restrictive food intake disorder. Ecological momentary assessment of ED behaviors during a two-week baseline assessment is used to personalize psychotherapy. Preliminary results in 79 patients suggest pre-post improvements in ED psychopathology following 10 sessions [109], but comparisons to active control have yet to be made.

Combining elements of studies on precision psychiatry [109], precision nutrition for cardiometabolic health [101], and deep meta-omic phenotyping of patients with AN can serve as a starting point for developing research designs that prioritize individualized weight restoration in AN. The Nutrition for Precision Health program enrolling 10,000 participants including 1,000 for outpatient controlled feeding and 500 for inpatient controlled feeding can serve as a model when developing studies for AN treatment [110]. Nutrition for Precision Health will generate multi-omic data sets and develop AI algorithms predicting individual responses to food, while leveraging data from large and diverse populations to ensure inclusivity and generalizability and avoid magnifying health inequities. To broaden the collection of studies with deep -omic phenotyping in AN, a completed study enrolled 255 participants with AN or controls without an ED to characterize associations of the gut microbiota with BMI before and after inpatient treatment, in tandem with experimental germ-free mouse models transplanted with the fecal microbiota of AN patients or controls [56]. This study and other genetic studies of AN [7] demonstrate the feasibility of enrolling large samples of participants with AN, which are needed to enable precision nutrition paradigms (NCT03119272). Broader interest from the community of ED researchers; external expertise from collaborators in nutrition science, microbiology, genetics, and advanced computational approaches; and investment from funding agencies are needed to galvanize the large multi-site studies required to carry out this work.

## Conclusion

The reviewed literature underscores the gaps in current weight restoration approaches for AN that may be remedied through incorporating precision nutrition. Key potential predictors of weight restoration to test in AI algorithms may include energy expenditure, fecal energy loss, gut microbiota composition and function, genetics, and psychiatric comorbidities. It remains unclear which of these factors, among others (i.e., metabolomics, proteomics) are the most critical to target. Other tailoring factors including participant demographics, clinical characteristics, and lifestyle behaviors may need to be considered. Research in this area is nascent but preliminary research suggests that personalizing inpatient protocols to factors such as AN duration may improve weight gain and enhance prognosis, with hints from observational studies that the gut microbiota may play a role in energy balance—a key determinant of weight status. In addition to a focus on calories, other elements of precision nutrition on which to tailor treatments could include macro- and micronutrient intake, and the timing, frequency, and distribution of meals, integrating flexibility to ensure patient acceptability particularly in outpatient settings. Ultimately, the clinical utility of bioinformatics approaches assimilating multi-omics data to predict optimal AN treatment depends on scalability at the population level (i.e., cost effectiveness), patient acceptability of algorithmically derived treatments, and translatability to the clinic. This narrative review serves as a call to action to investigate whether the recalcitrancy of AN to treatment can be combatted by personalizing nutrition therapies that augment weight restoration, minimize physiological and psychological distress, and reduce long-term relapse rates.

## Data Availability

No datasets were generated or analysed during the current study.

## Abbreviations

AN: Anorexia nervosa
ARFID: Avoidant/restrictive foodintake disorder
AI: Artificial intelligence
BMI: Body mass index
DNA: Deoxyribonucleic acid
ED: Eating disorder
EWAS: Epigenome-wide association studies
EPHX2: Epoxide hydrolase 2
FMT: Fecal microbiota transplantation
GI: Gastrointestinal
GWAS: Genome-wide association studies
ML: Machine learning
MTHFR: Methylenetetrahydrofolate reductase
NPH: Nutrition for precision health program
PUFA: Polyunsaturated fatty acid
POMC: Proopiomelanocortin
RMR: Resting metabolic rate
